# Synthesis and Properties of Poly(imides) and Poly(imides)/Ionic Liquid Composites Bearing a Benzimidazole Moiety

**DOI:** 10.3390/polym11050759

**Published:** 2019-04-30

**Authors:** Claudio A. Terraza, Pablo Ortiz, Luis H. Tagle, Germán Pérez, César Saldias, Fidel E. Rodríguez-González, Gustavo Cabrera-Barjas, Henry Catalán, Alain Tundidor-Camba, Deysma Coll

**Affiliations:** 1 Research Laboratory for Organic Polymers (RLOP), Faculty of Chemistry and of Pharmacy, Pontificia Universidad Católica de Chile, P.O. Box 306, Post 22, Santiago, Chile; cterraza@uc.cl (C.A.T.); ltagle@uc.cl (L.H.T.); geperezq@uc.cl (G.P.); ferg@uc.cl (F.E.R.-G.); hacatalan@uc.cl (H.C.); atundido@uc.cl (A.T.-C.); 2 UCEnergy Research Center, Pontificia Universidad Católica de Chile, Santiago, Chile; 3 Núcleo de Química y Bioquímica, Universidad Mayor, Santiago, Chile; pablo.ortiza@mayor.cl; 4 Chemistry-Physical Department, Faculty of Chemistry and of Pharmacy, Pontificia Universidad Católica de Chile, Santiago, Chile; cesaldiasb@gmail.com; 5 Unit for Technology Development (UDT), Universidad de Concepción, Av. Cordillera 2634, Parque Industrial Coronel, Concepción, Chile; g.cabrera@udt.cl

**Keywords:** poly(imides), benzimidazole, oxidative stability, ionic liquid

## Abstract

Three new aromatic poly(imides) containing benzimidazole units in the backbone were synthesized and characterized by several spectroscopic techniques. Flexible spacer groups were incorporated into the poly(imides) structure to improve their solubility in organic solvents and their oxidative stabilization. All poly(imides) were thermally stable (**T_d5%_** > 512 °C) and had the ability to form dense flexible films. Novel composite films were successfully prepared by loading poly(imide) with ionic liquid ([Bmim]Br) at different concentrations up to 25 wt.%. The resulting materials were characterized according to their morphology and elemental composition (SEM-EDX), water uptake capability, contact angle, and oxidative degradation resistance. Results suggested that poly(imide)/ionic liquid composites would be excellent candidates for future proton conductivity measurements.

## 1. Introduction

Poly(benzimidazoles) are polymers that contain a benzimidazole unit in their structure; the best-known member is poly(2,2’-*m*-(phenylene)-5,5’-bibenzimidazole) (*m*PBI). *m*PBI also has a high chemical and thermal resistance, with glass transition temperature (*T*_g_) values close to 450 °C [1,2,3,4,5,6]. The benzimidazole ring has the ability to absorb a large amount of acid when it is doped, thus favoring the formation of ionic clusters for proton transport [7]. These materials been used in Proton Exchange Membrane Fuel Cells (PEMFC), which is one of the most promising alternative power sources for portable uses. However, the oxidative degradation of PBI membranes is a key issue. Oxidative radicals like ·OH or ·OOH, which originate under PEMFC functioning, could attack the polymer hydrogen-containing bonds producing membrane degradation [8,9,10]. Some flexible spacer groups could enhance the oxidative stability of PBI membranes, such as fluoro-containing groups, sulfone, and ether linkage, among others [11,12,13,14].

The development of new oxidation-resistant polymers that could be used as membranes in PEMFC is strongly desired. Poly(imide)s (PIs) have been studied as one of the most promising candidates for that application due to their excellent chemical, thermal and mechanical stabilities, and good film-forming ability [15,16,17]. One method to improve oxidative stability in poly(imide)s-based membrane is by using monomeric diamines with linear configuration and flexible linkages [18]. However, the low solubility in common organic solvents limits their processing. The incorporation of flexible units and bulky groups such as –C(CF_3_)_2_– units and ether linkages improve the solubility (processability) of poly(imides) [12,14,19,20,21].

Previous investigations demonstrate the advantage of combining both the imide and the benzimidazole groups in the monomeric units to enhance the resulting polymer properties. Zhang et al. synthesized a poly(imide) containing a benzimidazole group, which showed good dimensional stability and proton conductivity [22]. Likewise, Chen et al. designed a novel PI-containing an imidazole ring, which had good proton conductivity at high temperature [23]. Therefore, a material that combines the advantages of PBI and PI is likely to exhibit excellent properties as a proton exchange membrane. However, these polymers require to be doped with a high acid content for maintaining proton conductivity, which decreases the stability of the membrane over time. In order to reduce or replace doping with acid, the use of ionic liquids (ILs) has been studied [24].

In previous studies, commercial Nafion® membranes have been doped with ionic liquids, giving them adequate values of ionic conductivity [25,26]. On another hand, composite membranes based on poly(imides)/ionic liquids and PBI/ionic liquids were synthesized, and an increase of the proton conductivity values of such materials was observed [27,28,29]. Ionic liquids are used like a solvent capable of proton conductivity, and it was observed that a proper pairing of the polymer and the IL is important for obtaining the excellent properties [30]. However, it is known that ionic liquids can degrade in the presence of free radicals much easier than polymeric materials [31], so the study of the oxidative stability of membranes containing ionic liquid must be carried out.

The present work reported the synthesis and characterization of three new poly(imides) containing benzimidazole group and flexible spacer groups along the polymer chain. These flexible spacer groups improved solubility and enhanced oxidative stability of the materials. Films composite based on these polymers and ionic liquids were prepared and characterized according to their thermal properties (TGA-DSC), morphology (SEM-EDX), water absorption, and oxidative stability.

## 2. Materials and Methods

### 2.1. Materials

4,4’-(Perfluoropropane-2,2-diyl)diphenol, 4,4’-sulfonyldiphenol, 4,4’-oxydiphenol, 4-nitrophthalonitrile and 4-nitrobenzene-1,2-diamine were obtained from AK Scientific Inc Company (San Francisco, USA). 4-Nitrobenzoyl chloride, Eaton’s reagent, anhydrous *N*,*N*-dimethylformamide (DMF), anhydrous *N,N*-dimethylacetamide (DMAc), anhydrous pyridine (Py), acetic anhydride (Ac_2_O) and 1-butyl-3-methylimidazolium bromide ([Bmim]Br) were obtained from Sigma-Aldrich-Merck Company (Milwaukee, WI, USA). 2-(4-Aminophenyl)-*1H*-benzimidazole-5-amine (*p*-DABI) was synthesized according to the literature [32]. All other reagents and solvents were purchased commercially as analytical-grade and used without further purification.

### 2.2. Instrumentation

FT-IR-ATR (LiTaO_3_) were recorded on Perkin Elmer Spectrum Two (Connecticut, USA), spectrophotometer over the range of 8300–350 cm^−1^. ^1^H and ^13^C NMR spectra were carried out on a 400 MHz instrument (Bruker AC-200 Bremen, Germany) using DMSO-*d_6_* as solvent and TMS as an internal standard. Melting points were obtained on a SMP3 Stuart Scientific melting point apparatus (Stafford, United Kingdom). Glass transition temperature (*T*_g_) values were obtained with a Mettler-Toledo (Greifensee, Switzerland) DSC 821 calorimetric system from the second run (10 °C/min under N_2_ flow). Thermogravimetric analyses (TGA) were carried out in a Mettler (Switzerland, Switzerland) TA-3000 calorimetric system equipped with a TC-10A processor and a TG-50 thermobalance with a Mettler MT5 microbalance (temperature range between 25 °C and 900 °C at 10 °C/min under N_2_ flow). Viscosimetric measurements were made in a Desreux-Bischof type dilution viscosimeter using at 30 ± 0.1 °C (c = 0.5 g/dL). Elemental analysis was made on a Fisons EA 1108-CHNS-O equipment (Thermo Scientific, Waltham, MA, USA). A Dataphysics OCA 20 device with a conventional goniometer and high-performance video camera, controlled by SCA20 software were used to measure the optical contact angle (DataPhysics, Filderstadt, Germany). Morphological analysis was performed with an ETEC autoscan Model U-1 scanning electron microscope couple to JeoL EDS device (University of Massachusetts, Worcester, MA, USA). The samples were fixed in a sample holder and covered with a gold layer for 3 min using an Edwards S150 sputter coater (BOC Edwards, São Paulo, Brazil) before analysis.

### 2.3. Synthesis of Tetranitrile Precursors (4, 5 and 6)

To a round bottom flask were added 9 mmol of the corresponding bisphenol (4,4’-(perfluoropropane-2,2-diyl)diphenol, 4,4’-sulfonyldiphenol or 4,4’-oxydiphenol), 18 mmol of 4-nitrophthalonitrile, 36 mmol of potassium carbonate and 40 mL of DMF. The mixture was heated at 60 °C for 24 h under nitrogen atmosphere. After that, the suspension was poured into 300 mL of deionized water with stirring. The resulting solid was filtered, washed with methanol and dried at 60 °C for 12 h.


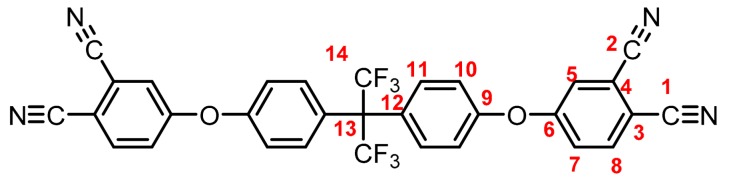


**(4): Yield: 98%.** M.p.: 225-227 °C. IR (KBr, ν, cm^−1^): 3086 (C-H arom.); 2237 (CN); 1589, 1489 (C=C); 1200 (C-O-C); 1087 (C-F) 848 (arom. *p*-disubst.). ^1^H NMR (400.13 MHz, DMSO-*d_6_*, δ, ppm): 8.17 (d, *J = 8.7 Hz,* 2H, (H8)); 7.96 (d, *J = 1.9 Hz,* 2H, (H5)); 7.56 (d, *J = 8.7 Hz*, 2H, (H7)); 7.51 (d, *J = 8.4 Hz,* 4H, (H11)); 7.33 (d, *J = 8.7 Hz*, 4H, (H10)). ^13^C NMR (100.62 MHz, DMSO-*d_6_*, δ, ppm): 159.4 (C6); 154.4 (C9); 135.9 (C11); 131.6 (C8); 128.3 (C12); 124.8, 121.9 (C14); 123.1 (C7); 122.0 (C5); 119.4 (C10); 116.4 (C4); 115.3 (C1); 114.8 (C2); 108.7 (C3); 63.9 (C13). Elem. Anal. Calcd. for C_31_H_14_F_6_N_4_O_2_ (588.10): C, 63.27%; H, 2.40%; F, 19.37%; N, 9.52%; O. 5.44%. Found: C, 63.21%; H, 2.35%; F, 19.22%; N, 9.61%; O. 5.41%.


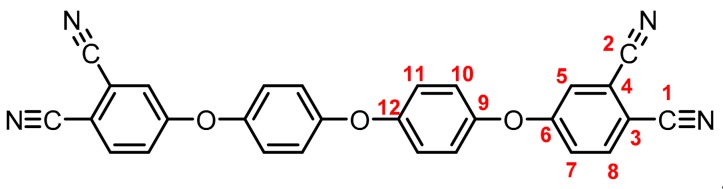


**(5):** Yield: 96%. M.p.: 228-231 °C. IR (KBr, ν, cm^−1^): 3086 (C-H arom.); 2237 (CN); 1589, 1489 (C=C); 1205 (C-O-C); 848 (arom. *p*-disubst.). ^1^H NMR (400.13 MHz, DMSO-*d_6_*, δ, ppm): 8.11 (d, *J = 8.8 Hz,* 2H, (H8)); 7.79 (d, *J = 2.1 Hz,* 2H, (H5)); 7.43 (dd, *J = 8.7, 2.2 Hz*, 2H, (H7); 7.27 (d, *J = 8.9 Hz,* 4H, (H11)); 7.21 (d, *J = 9.0 Hz*, 4H (H10)). ^13^C NMR (100.62 MHz, DMSO-*d_6_*, δ, ppm): 160.9 (C6); 153.6 (C9); 148.6 (C12); 135.7 (C8); 121.7 (C10); 121.6 (C7); 121.1 (C5); 120.0 (C11); 116.1 (C4); 115.3 (C1); 114.8 (C2), 107.5 (C3). Elem. Anal. Calcd. for C_28_H_14_N_4_O_3_ (454.11): C, 74.0%; H, 3.11%; N, 12.33%; O. 10.56%. Found: C, 73.87%; H, 3.05%; 12.35%; O. 10.51%.


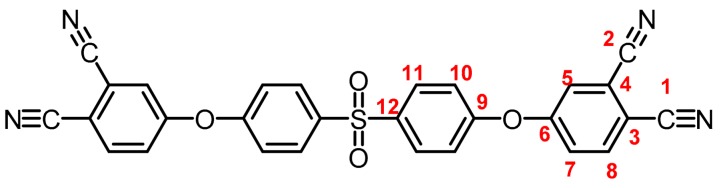


**(6):** Yield: 97%. M.p.: 206-209 °C. IR (KBr, ν, cm^−1^): 3093 (C-H arom.); 2237 (CN); 1589, 1489 (C=C); 1203 (C-O-C); 1149 (S=O); 840 (arom. *p*-disubst.); 732, 686 (arom. trisubst.). ^1^H NMR (400.13 MHz, DMSO-*d_6_*, δ, ppm): 8.18 (d, *J = 8.7 Hz,* 2H, (H8)); 8.08 (d, *J = 8.5 Hz,* 4H, (H11)); 8.01 (s, 2H, (H5)); 7.62 (d, *J = 8.6 Hz,* 2H, (H7)); 7.38 (d, *J = 8.4 Hz*, 4H (H10)). ^13^C NMR (100.62 MHz, DMSO-*d_6_*, δ, ppm): 158.9 (C6); 158.6 (C9); 137.1 (C12); 136.4 (C8); 130.3 (C11); 124.4 (C7); 124.2 (C5); 120.1 (C10); 116.9 (C4); 115.6 (C2); 115.2 (C1), 109.9 (C3). Elem. Anal. Calcd. for C_28_H_14_N_4_O_4_S (502.50): C, 66.93%; H, 2.81%; N, 11.15%; O, 12.74%; S, 6.38%. Found: C, 66.89%; H, 2.82%; N, 11.07%; O, 12.65%; S, 6.39%.

### 2.4. Synthesis of Tetracarboxylic Acid Precursors (7, 8 and 9)

To a three-neck bottom flask equipped with a condenser and a magnetic stirrer bar were added the tetranitrile precursor (6 mmol), a mixture of ethanol (50 mL) and an aqueous potassium hydroxide solution (1.5 M) (50 mL). The reaction mixture was heated under reflux for 72 h (time that ammonia emission ceased). The mixture was cooled at room temperature, filtered through a gravity funnel to eliminate any suspension and diluted with 50 mL of water. The resulting mixture was cooled by an ice-water bath and was acidified until pH 3 with an aqueous solution of HCl (6N). The solid formed was filtered under reduced pressure, washed thoroughly with water and dried in an oven at 60 °C for 12 h.


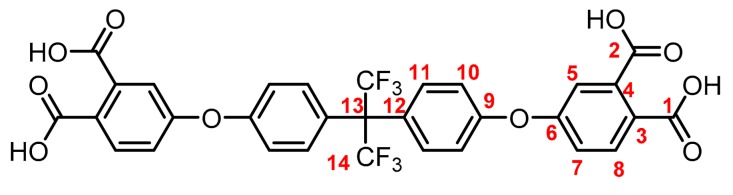


**(7): Yield: 92%.** M.p.: >300 °C. IR (KBr, ν, cm^−1^): 3510 (OH); 3078 (C–H arom.); 1789, 1697 (C=O); 1604 (C=C); 1201 (C–O–C); 1072 (C-F); 833 (arom. *p*-disubst.). ^1^H NMR (400.13 MHz, DMSO-*d_6_*, δ, ppm): 7.86 (d, *J = 8.5 Hz,* 2H, (H8)); 7.46 (d, *J = 8.4 Hz,* 4H, (H11)); 7.34 (d, *J = 1.4 Hz*, 2H, (H5)); 7.28 (s, *2*H, (H7)); 7.23 (d, *J = 8.7 Hz*, 4H, (H10)). ^13^C NMR (100.62 MHz, DMSO-*d_6_*, δ, ppm): 168.7 (C1); 168.0 (C2) 158.2 (C6); 156.9 (C9); 137.0 (C4); 132.23 (C8); 132.18 (C11); 128.1 (C3); 125.9 (C12), 123.0 (C14); 120.7 (C7); 119.4 (C5); 118.8 (C10); 63.8 (C13). Elem. Anal. Calcd. for C_31_H_18_F_6_O_10_ (664.47): C, 56.04%; H, 2.73%; F, 17.16%; O. 24.08%. Found: C, 56.12%; H, 2.61%; F, 17.115%; O. 24.17%.


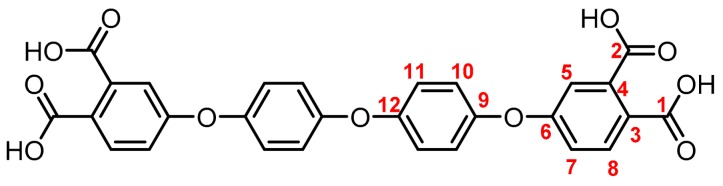


**(8):** Yield: 91%. M.p.: > 300 °C. IR (KBr, ν, cm^−1^): 3479 (OH); 1790, 1728 (C=O); 1604 (C=C); 1207 (C–O–C); 879 (arom. *p*-disubst.); 786 (arom. trisubst.). ^1^H NMR (400.13 MHz, DMSO-*d_6_*, δ, ppm): 7.79 (d, *J = 8.9 Hz,* 2H, (H8)); 7.23–7.12 (m, 12H, (H7, H5, H10, H11)); ^13^C NMR (100.62 MHz, DMSO-*d_6_*, δ, ppm): 169.0 (C1); 167.9 (C2), 160.2 (C6); 154.0 (C9); 150.9 (C12); 137.1 (C4); 131.9 (C8); 125.9 (C3); 122.3 (C10); 120.8 (C11); 118.7 (C7); 116.5 (C5). Elem. Anal. Calcd. for C_28_H_18_O_11_ (530.44): C, 63.40%; H, 3.42%; O. 33.18%. Found: C, 63.42%; H, 3.38%; O. 33.09%.


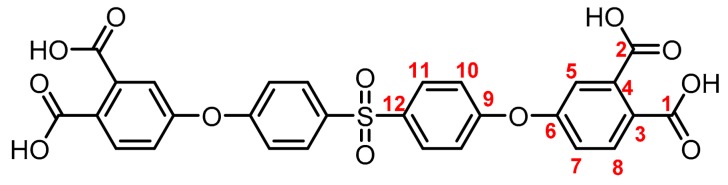


**(9):** Yield: 92%. M.p.: > 300 °C. IR (KBr, ν, cm^−1^): 3587 (OH); 1743, 1738 (C=O); 1489 (C=C); 1203 (C-O-C); 1150 (S=O); 888 (arom. *p*-disubst.). ^1^H NMR (400.13 MHz, DMSO-*d_6_*, δ, ppm): 8.0 (d, *J = 8.8 Hz,* 4H, (H11)); 7.81 (d*, J = 8,5 Hz, 2*H, (H8); 7.33 (d, *J = 2.4 Hz*, 2H, (H5)); 7.28 (dd, *J = 8.5, 2,5 Hz*, 2H, (H7)); 7.25 (d, *J = 8.8 Hz*, 4H, (H10)) ^13^C NMR (100.62 MHz, DMSO-*d_6_*, δ, ppm): 169.0 (C1); 168.0 (C2), 160.6 (C9); 157.2 (C6); 136.9 (C12); 136.6 (C4); 132.0 (C8); 130.57 (C11); 130.56 (C7);128.6 (C3); 121.7 (C5); 119.6 (C10). Elem. Anal. Calcd. for C_28_H_18_O_12_S (578.50): C, 58.13%; H, 3.14%; O, 33.19%; S, 5.54% Found: C, 59.00%; H, 3.17%; O, 33.02%; S, 5.49%.

### 2.5. Synthesis of Tetracarboxylic Anhydride Monomers (10, 11 and 12)

To a round bottom flask equipped with a condenser and a magnetic stirrer bar were added 10 mmol of tetracarboxylic acid precursor and 25 mL of acetic anhydride. The reaction mixture was heated under reflux for 12 h under nitrogen atmosphere. After this time, the reaction was cooled at room temperature and a white solid was formed. The solid was filtered under reduced pressure and dried at 200 °C for 12 h.


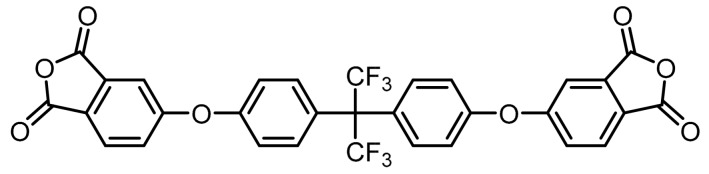


**(10):** Yield: 85%. M.p.: 227–231 °C. IR (KBr, *ʋ*, cm^−1^): 3101 (C–H arom.); 1843, 1782 (C=O); 1512 (C=C); 1200 (C–O–C); 1072 (C–F), 887 (arom. *p*-disubst.). Elem. Anal. Calcd. for. C_31_H_14_F_6_O_8_; (628.44): C, 59.25%; H, 2.25%; F, 18.14%; O, 20.37%. Found: C, 59.13%; H, 2.32%; F, 17.99%; O, 20.35%.


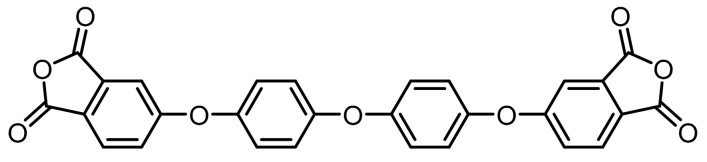


**(11):** Yield: 83%. M.p.: 239–241 °C. IR (KBr, *ʋ*, cm^−1^): 3101 (C–H arom.); 1843, 1774 (C=O) 1597 (C=C); 1207 (C–O–C); 887 (arom. *p*-disubst.). Elem. Anal. Calcd. for. C_28_H_14_O_9_; (494,41): C, 68.02%; H, 2.85%; O, 29.12%; Found: C, 68.21%; H, 2.91%; O, 29.01%.


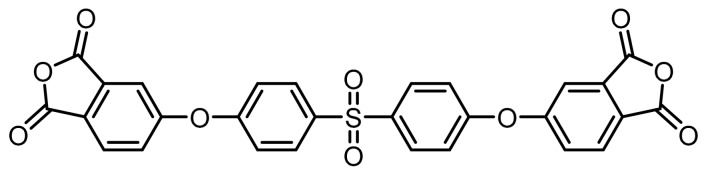


**(12):** Yield: 86%. M.p.: 245–247 °C. IR (KBr, *ʋ*, cm^−1^): 3101 (C–H arom.); 1843, 1789 (C=O); 1582 (C=C); 1203 (C–O–C); 1149 (S=O), 887 (arom. *p*-disubst.). Elem. Anal. Calcd. for. C_28_H_14_O_10_S; (542.47): C, 62.00%; H, 2.60%; O, 29.49%; S, 5.91% Found: C, 62.07%; H, 2.50%; O, 28.99%; S, 6.01%.

### 2.6. Synthesis of 2-(4-Aminophenyl)-1H-benzimidazole-5-amine (p-DABI)

Diamine was synthesized according to the literature [32]. Briefly, 4-nitrobenzene-1,2-diamine and 4-nitrobenzoyl chloride were dissolved in Eaton’s reagent and heated at 120 °C overnight. The resulting solution was poured onto water and the precipitate was separated by filtration to give the dinitro benzimidazole derivative, which was heated at reflux in ethanol in presence of Pd/C (10%) and hydrazine monohydrate (80%). After that, the mixture was cooled to room temperature, Pd/C was filtered off and the filtrate was poured onto water and neutralized with an aqueous solution of HCl to give a solid, which was recrystallized from an ethanol/water (1:1 *v*/*v*) mixture.

### 2.7. Synthesis of Poly(imides) (PI-F, PI-O and PI-S)

All polymers were prepared following the general method described below: in a three-necked round bottom flask equipped with mechanical stirrer and nitrogen atmosphere inlet and outlet, 2.0 mmol of *p*-DABI in 8 mL of DMAc were suspended. Then, 2.0 mmol of the corresponding tetracarboxylic anhydride was added at once in solid form and stirred at room temperature for 6 h. During this time, the suspension originates a viscous solution. After that, 1.0 mL of acetic anhydride and 0.8 mL of pyridine were added, and the mixture was stirred for two hours at room temperature and subsequently one hour at 60 °C. Then, the mixture was cooled at room temperature and poured on 500 mL of deionized water with vigorous stirring. The obtained fibers were washed with methanol and dried at 150 °C for 12 h.


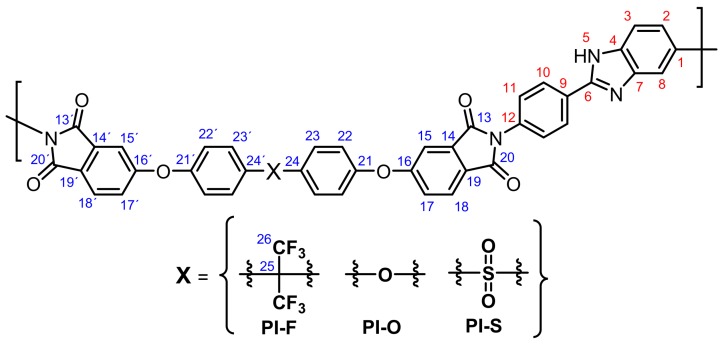


**(PI-F):** FT-IR-ATR (film, ν, cm^−1^): 3072, 2975, 2924 (C–H); 1775, 1712 (C=O); 1618 (C=N); 1602, 1508, 1477 (C=C); 1365 (C–N); 1258, 1076 (C–O–C); 1235, 1170 (C-F); 834, 810,780, 742 (C–H, out of plane). ^1^H NMR (400,13 MHz, DMSO-d_6_, δ ppm): 13.33 (broad peak, 1H, (H5)); 8.34 (d, *J* *= 8.0 Hz*, 2H, (H10)); 8.03 (broad peak, 2H, (H18)); 7.75 (broad peak, 1H, (H3)), 7.71(s, 1H, (H8)); 7.67 (d, *J* *= 8.0 Hz*, 2H, (H11)); 7.58 (s, 2H, (H15)); 7.56 (broad peak, 2H, (H17)); 7.52 (d, *J* *= 8.3 Hz*, 4H, (H23)); 7.32 (d, *J* *= 8.4 Hz*, 4H, (H22)); 7.27 (d, *J* *= 8.5 Hz*, 1H, (H2)). ^13^C NMR (100.62 MHz, DMSO-d_6_, δ ppm): 166.75, 166.59 (C20, C20’); 166.10, 165.96 (C13, C13’); 161.46, 161.28 (C16, C16’); 156.13, 156.03 (C21, C21’); 151.96 (C6); 134.46 (C7); 134.33 (C12); 133.31 (C4); 131.99, 132.00 (C23, C23’); 129.28 (C1); 128.30, 128.22 (C24, C24’); 127.51 (C10); 127.05 (C11); 126.57, 126.59 (C14, C14’); 126.36, 126.33 (C17, C17’); 126.24 (C9); 126.10, 125.91 (C18, C18’); 124.21, 124.09 (C19, C19’); 124.00 (q, *J* = 287.5 Hz, C26); 122.07 (C2); 119.57, 119.50 (C22, C22’); 118.97 (C8); 114.87 (C3); 113.23, 113.15 (C15, C15’); 63.41 (hept, *J* = 25.0 Hz, C25).

**(PI-O):** FT-IR-ATR (film, ν, cm^−1^): 3080, 2973, 2924 (C–H); 1775, 1712 (C=O); 1617 (C=N); 1605, 1490, 1475 (C=C); 1364(C–N); 1273, 1226, 1077 (C–O–C); 834, 808,778, 743 (C–H, out of plane). ^1^H NMR (400,13 MHz, DMSO-d_6_, δ ppm): 13.21 (broad peak, 1H, (H5)); 8.33 (d, *J* *= 8.4 Hz*, 2H, (H10)); 7.99 (broad peak, 2H, (H18)); 7.74 (broad peak, 1H, (H3)); 7.70 (s, 1H, (H8)); 7.65 (d, *J* *= 8.4 Hz*, 2H, (H11)); 7.46 (d, *J* *= 7.8 Hz*, 2H, (H17); 7.40 (s, 2H, (H15)); 7.26 (broad peak, 4H, (H22)); 7.22 (broad peak, 5H, (H23), (H2)). ^13^C NMR (100.62 MHz, DMSO-d_6_, δ ppm): 166.81, 166.69 (C20, C20’); 166.16, 166.06 (C13, C13’); 163.24, 163.09 (C16, C16’); 153.92, 153.88 (C21, C21’); 151.94 (C6); 150.22, 150.14 (C24, C24’); 134.36 (C7); 134.22 (C12); 133.32 (C4); 129.23 (C1); 127.48 (C10); 127.02 (C11); 126.26 (C9); 125.99, 125.80 (C18, C18’); 125.38, 125.27 (C14, C14’); 125.22, 125.17 (C19, C19’); 122.66, 122.55 (C17, C17’); 122.16 (C2); 122.12, 122.09 (C22, C22’); 120.52, 120.45 (C23, C23’); 118.96 (C8); 114.72 (C3); 111.29, 111.22 (C15, C15’).

**(PI-S):** FT-IR-ATR (film, ν, cm^−1^): 3080, 3066, 2973, 2953 (C-H); 1776, 1712 (C=O); 1618 (C=N); 1584, 1487, 1475 (C=C); 1364 (C–N); 1265, 1071 (C-O-C); 1145 (S=O); 834, 810,785, 743 (C–H, out of plane). ^1^H NMR (400,13 MHz, DMSO-d_6_, δ ppm): 13.27 (broad peak, 1H, (H5)); 8.34 (d, *J* *= 7.9 Hz*, 2H, (H10)); 8.07 (d, *J* *= 8.5 Hz*, 4H, (H23)); 8.04 (broad peak, 2H, (H18)); 7.75 (broad peak, 1H, (H3)); 7.71 (s, 1H, (H8)); 7.66 (two peak, 4H, (H11, H15)); 7.59 (d, *J* *= 8.0 Hz*, 2H, (H17)); 7.34 (d, *J* *= 8.3 Hz*, 4H, (H22)); 7.27 (d, *J* *= 8.2 Hz*, 1H, (H2)). ^13^C NMR (100.62 MHz, DMSO-d_6_, δ ppm): 166.68, 166.48 (C20, C20’); 166.03, 165.85 (C13, C13’); 160.40, 160.22 (C21, C21’); 159.94, 159.84 (C16, C16’); 151.96 (C6); 136.60, 136.53 (C24, C24’); 134.49 (C7); 134.36 (C12); 133.27 (C4); 130.26, 130.15 (C23, C23’); 129.31 (C1); 127.52 (C10); 127.38, 127.25 (C14, C14’); 127.18, 127.09 (C19, C19’); 127.05 (C11); 126.20 (C9); 126.13, 125.94 (C18, C18’); 125.26, 125.15 (C17, C17’); 125.15 (C17); 122.06 (C2); 119.55, 119.47 (C22, C22’); 119.07 (C8); 114.83 (C3); 114.42, 114.36 (C15, C15’).

### 2.8. Dense Films Preparation

PI dense films were prepared by casting method from a solution of 500 mg of each polymer in 10 mL of DMF. The solution was filtered using a syringe filter of 3.1 µm of pore size over a Teflon plate of 5 cm diameter, which was subsequently placed on a heating plate at 60 °C for 12 h. The film was removed and placed in a stainless-steel mesh to be treated at 200 °C for 24 h. Poly(imide)/ionic liquid (PI/IL) composite dense films were prepared following the same methodology described above but adding 25 weight% of 1-butyl-3-methylimidazolium bromide ([Bmim]Br) to the solution.

### 2.9. Water Uptake Measurements

Water absorption properties of pure PI and PI/IL composite dense films were measured by weighing the previously dry films and immersed them in deionized water for 24 h at 30 °C and at 80 °C. Films were removed, wiped with tissue paper and weighed again. This procedure was performed five times to ensure an average measurement [33].

### 2.10. Oxidative Stability

One hundred milligrams of dense films (pristine PI and PI/IL) were totally immersed in Fenton’s solution, composed of 3 wt.% H_2_O_2_ aqueous solution and 3 ppm of FeSO_4_ at 80 °C. The oxidative stabilities were obtained by the elapsed time before films started to become a slightly brittle, and the degradation was recorded in terms of the weight loss at that time.

## 3. Results and Discussion

### 3.1. Monomers Synthesis and Characterization

**Figure 1:**
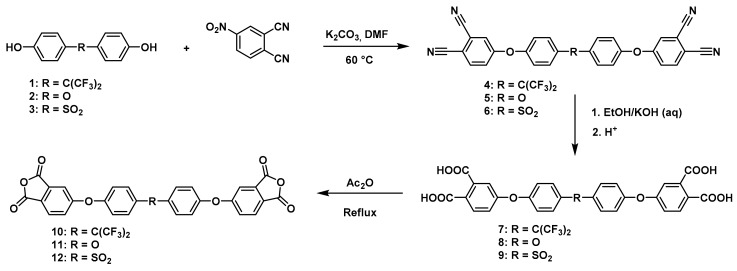
Synthetic route of precursors (**4**–**9**) and tetracarboxylic dianhydride monomers (**10**–**12**).

The synthesis of tetracarboxylic anhydride monomers (**10**–**12**) was carried out according to the literature [34]; it consisted in three reaction stages (Figure 1). Firstly, an aromatic nucleophilic substitution reaction between diphenol (**1**–**3**) and 4-nitrophthalonitrile in presence of potassium carbonate gave the tetranitrile derivatives. An important key in this step is the temperature of reaction, which must does not exceed 60 °C because the reaction changes from a red to an intense black color, indicating the presence of collateral reactions [35], which have been explained and studied by Parker et al. [36,37]. The second step consists in the transformation of the tetranitrile compounds (**4**–**6**) into the corresponding tetracarboxylic acids by alkaline hydrolysis, using potassium hydroxide in a mixture of ethanol-water. In general, this reaction can be monitored by detecting the gaseous ammonia putting an acidity indicator paper at the outlet of the refrigerant. Once the indicator paper does not detect the ammonia emanation, the reaction is stopped and brought to pH 3 by adding an aqueous HCl solution.

The last stage of the route consisted of a cyclodehydration reaction using acetic anhydride as a dehydrating agent. The product obtained was isolated by filtration under reduced pressure since on cooling the reaction mixture the compound precipitated in the form of high purity crystals.

The structure of the compounds (**4**–**12**) was confirmed by elemental analysis, FT-IR and NMR spectroscopies (see Material and Methods section). The FT-IR spectra of the tetranitrile derivatives (**4**–**6**) showed the characteristic intense signal of the C–N stretch of the nitrile group around 2237 cm^−1^. That signal was not observed in the spectra of the tetracarboxylic acids (**7**–**9**), where the stretching of the O–H bond around 3500 cm^−1^ and the C–O bond of the carbonyl group around 1720 cm^−1^ were evidenced. In the spectra of the tetracarboxylic dianhydride monomers (**10**–**12**) the O–H stretching was not observed, and the symmetric and asymmetric stretching pattern of the anhydride carbonyls were observed around 1840 and 1780 cm^−1^, respectively. In the ^1^H NMR is possible to see a similar chemical shift for the protons of the precursors **4**–**6**. Protons belonging to the ring that contain the two nitrile groups (H5, H7, and H8) were more shifted that protons H10 and H11 due to the electro withdrawing effect of these groups, except for the compound **6**. Thus, the sulfonyl group in *ortho* position to H11 promotes an electro withdrawing effect, which removes electronic density by shifting H11 to a low magnetic field. When the –CN groups are replaced by –COOH groups in **7**–**9**, the electro withdrawing effect of the ring decreases and the chemical shifts of the protons H5, H7, H8 and H11 are located close to each other, depending on the different shielding effects caused by the groups –CF_3_, –O– or –SO_2_ on *ortho*-position with respect to H11.

### 3.2. Poly(imides) Synthesis

The usual method for the preparation of aromatic PIs consisted of two stages. The first one was the formation of the poly(amic acid) from the reaction between the diamine (*p*-DABI) and the corresponding tetracarboxylic anhydride in DMAc at room temperature for six hours. The second step was an intramolecular cyclodehydration to generate the final poly(imide) using pyridine and acetic anhydride as condensing agents.

Due to the low solubility of the diamine monomer in the polymerization solvent (DMAc, or NMP), several tests for the preparation of the poly(amic acids) were carried out. These tests consisted in to add lithium chloride to improve the solubility of the diamine on the reaction solvent (DMAc) or incorporate chlorotrimethylsilane (TMSCl) to increase the nucleophilic of the diamine, which also improved the solubility [38]. Table 1 summarizes the reaction conditions for the synthesis of PI-F taking account the obtained viscosity for each attempt.

The diamine was dissolved when lithium chloride was added to the reaction mixture in attempt 1 (4 mmol total of monomer in 4 mL of solvent) and attempt 2 (4 mmol total of monomer in 8 mL of solvent) but the inherent viscosity results did not exceed 0.35 dL/g. The use of TMSCl has been reported previously to increase the nucleophilic character of the diamine and in our case, this additive also solubilized the diamine allowing the polymerization to occur in homogeneous medium. By adding TMSCl it was possible to obtain polymers with higher viscosity values (attempt 3 and 4) than by using LiCl. However, the use of molar concentration of 0.5 M (using 8 mL of solvent) favored the increase of the molecular weight. In the last attempt, a molar concentration of 0.5 M was employed without any additive to lead the polymerization reaction to occur in a heterogeneous medium. In that case, a completely homogeneous solution was obtained after two hours increasing their viscosity over time. Using this experimental methodology was possible to obtain poly(imides) with a high molecular weight (inherent viscosity of 0.72 dL/g).

**Figure 2:**
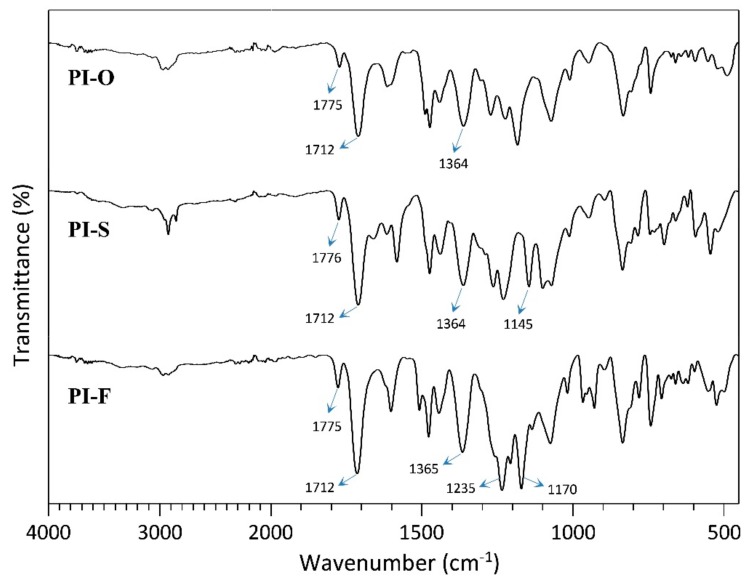
FT-IR spectra for the poly(imides).

The chemical structure for each newly synthesized poly(imides) was confirmed by spectroscopic methods. In FT-IR spectra, the successful conversion of poly(amic acid) to PI was confirmed by the characteristic imide group absorption bands observed around 1775 and 1712 cm^−1^, which correspond to the C=O symmetric and asymmetric stretching vibrations, respectively (Figure 2). Additionally, it was corroborated by the absence of a wide band between 3600 and 3200 cm^−1^ corresponding to N–H and O–H stretching vibrations. The bands of the S=O stretching vibrations (around 1145 cm^−1^) for PI-S and C–F (around 1235 and 1170 cm^−1^) for PI-F were also observed in their particular FT-IR spectra.

**Figure 3:**
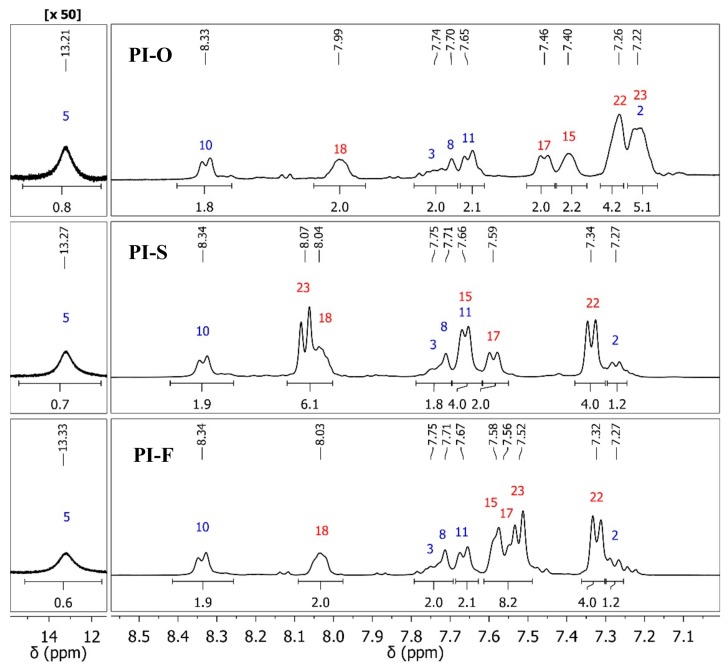
Selected region of the ^1^H NMR spectra (400 MHz, DMSO-*d_6_*) for PIs.

Results of the characterization by solution ^1^H and ^13^C NMR techniques also confirmed the expected structure for each poly(imide). Figure 3 shows selected regions from the ^1^H-NMR spectra where all the hydrogen signals of these poly(imides) were assigned. The protons chemical shifts from the diamine moiety (H2, H3, H5, H8, H10 and H11) are practically equal for all polymers because these protons are located far from the X group coming from the fragment provided by the dianhydride monomer, which differentiates each poly(imide). The main difference obtained in ^1^H NMR spectra chemical shifts were between the protons H23 of each polymer, due to the different shielding effects caused by the X groups on *ortho*-position with respect to these nuclei.

Similar behavior was observed in ^13^C NMR spectra (see Material and Methods section); most differences in chemical shifts appeared among carbon nuclei close to the group X (C24, C21, C22 and C21). In the case of PI-F, with C(CF_3_)_2_ as central element, the multiplets due to the 2JC-F and 3JC-F couplings corresponding to the carbon nuclei C26 (*q*, *J* = 287.5 Hz) and C25 (*hept*, *J* = 25.0 Hz), respectively, were also detected. The detection of duplicate signals for carbon atoms of the fragments provided by the dianhydride monomers could be due to the two possible chemical environments generated by the asymmetry of the diamine.

### 3.3. Polymers solubility and Inherent Viscosity

The solubility of the poly(imides) was determined in different common organic solvents at room temperature and by heating the sample to each solvent boiling point temperature (Table 2). The behavior of all PIs was similar, showing high solubility at room temperature in polar aprotic solvents such as DMSO, DMF, DMAc, and NMP. In addition, the polymers were soluble in *m*-cresol (under heating). PI-F, despite the high aromatic content that can be appreciated in their structure, was soluble, even in THF. This fact could be associated with the great volume of CF_3_ groups, which generate spaces between the chains, facilitating solvation, swelling and subsequent solubilization of the material.

It is important to highlight that most of the previously reported synthetic poly(imide)s without flexible spacer groups were insoluble in such organic solvents [39,40,41].

In accordance with the previous studies showed in Table 1, the inherent viscosity values for all poly(imides) were similar and high enough to ensure that medium-to-high molecular weights had been achieved. Molecular weights were not measured but it was high enough to prepare good quality tactile resistance dense films.

### 3.4. Poly(imide) and Poly(imide)/ionic Liquid Dense Film Preparation

PIs and poly(imide)/ionic liquid (PI/IL) composite dense films were prepared following the procedure described in Section 2.8. All PIs dense films were resistant to handling, showing an excellent quality film. In order to choose the optimal amount of [Bmim]Br, the quality of the film was analyzed at different concentrations of the IL (10, 15, 20, 25, 30, 35 and 40 weight%). [Bmim]Br was chosen due to the similitude of the cation structure (imidazolium) with the diamine structure (benzimidazole), and its cheap price. PI-F polymer was chosen to prepare the composite samples due its good solubility and high molecular weight. After final thermal treatment (200 °C for 24 h), the films were tested manually in relation to their mechanical strength and the results are summarized in Table 3.

Due to the nonvolatile nature and thermal stability of IL, they can be used as plasticizers, as well as suppliers of free charge carriers for novel solid polymer electrolyte film preparation [42]. It should be noted the maximum ionic liquid loading that allows obtaining flexible and dense film was 25 wt.% (PI-F/IL_25_), confirming the plasticizer effect of IL at low concentration. At over 25% content of IL, brittle films were obtained; this fact could be related to the potential reduction in the chain interactions inside the polymeric system.

### 3.5. Thermal Properties of PI and PI/IL Films

**Figure 4:**
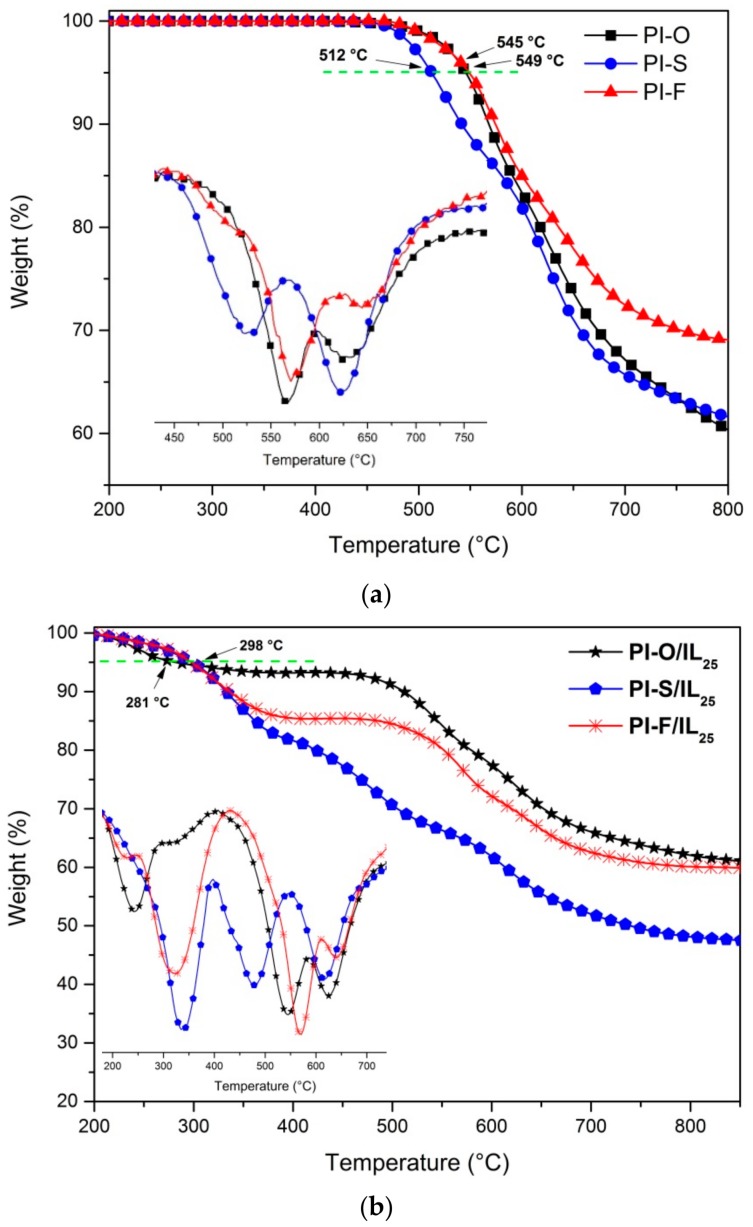
TGA and DTGA curves of the synthesized PIs (**a**) and PI/IL films (**b**).

The thermal properties of the PI films were studied under nitrogen atmosphere by thermogravimetric analysis (TGA and DTGA) and differential scanning calorimetry (DSC), while PI/IL films were evaluated only by TGA and DTGA analysis. The curves are shown in Figure 4 and the data are summarized in Table 4.

The results indicated that all PIs were thermally stable with decomposition temperatures (*T*_d5%_) higher than 512 °C. This great thermal stability could be attributed to the high aromatic content on the main chain of the polymers. The DTGA curve indicates two important weight losses in each polymer; the first is around 520–570 °C, probably associated with simultaneous depolymerization reaction, rupture, and volatilization of fragments related to the central group on each dianhydride segment and other labile single bonds. The second one is around 620–650 °C, which could be associated with the complete decomposition of the polymer aromatic chains.

The 5% weight loss temperature of all PI/IL films was lower than the pristine PIs, all of them close to 300 °C. This fact is related to the presence of IL, which decomposes at 273 °C [43]. In the DTGA graph of the PI/IL films, a first loss between 250–350 °C was clearly observed. The decomposition values of the ionic liquid were shifted to higher values probably because the ionic liquid was protected by the polymer chains. All PI/IL films demonstrated great thermal properties compared to the PBI/H_3_PO_4_ system, which is used in PEMFC and it degraded at 160 °C [44].

The *T*_g_ values were obtained only for PI films and were in the range of 225–260 °C, and there was a tendency to decrease the *T*_g_ when the flexibility of the central group increased. Thus, PI-F has the highest value due to the rigidity of the –C(CF_3_)_2_– moiety in comparison to –O– and –SO_2_–. This behavior has been reported for poly(imides) that contain a dianhydride with –C(CF_3_)_2_– groups in their structures [45,46,47,48,49]. According to the high thermal stability and *T*_g_ values showed for all PIs, the new materials would be suitable for application at high-temperature, by including proton exchange membrane.

### 3.6. SEM-EDX Analysis

**Figure 5:**
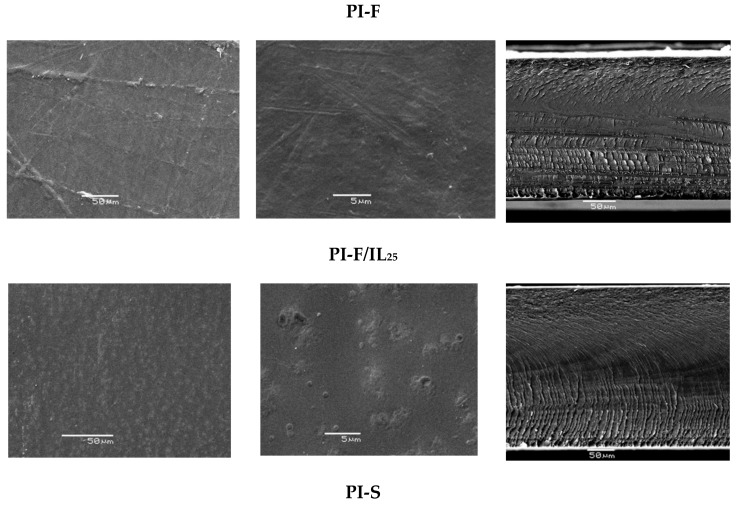

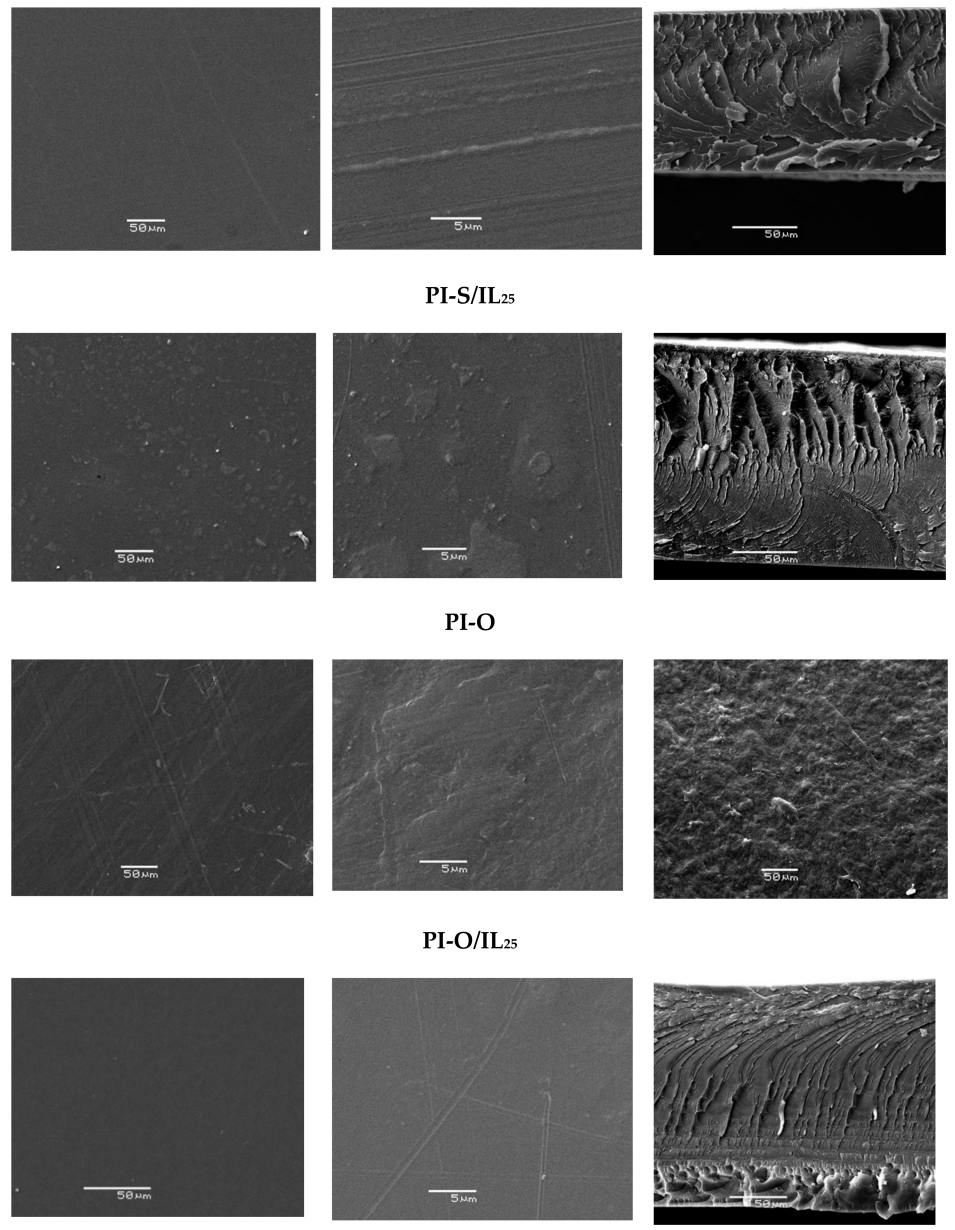
SEM micrographs of the PIs films and PI/IL_25_ composite films. Left and central columns: surface micrographs. Right column: side view micrographs.

Figure 5 shows the SEM micrographs of the PIs films and PI/IL_25_ composite films. As can be seen, PI-F, PI-S, and PI-O films showed a fibrous surface morphology, while PI-F-IL_25_ and PI-S-IL_25_ have a continuous surface with the presence of some agglomerates. These differences are probably due to the presence of [Bmim]Br, which would interact with the polar groups of the polymer chains, producing aggregates that would spread on the surface during the manufacturing process of the membranes. In the case of PI-O/IL_25_, their SEM micrographs showed a free-agglomerates surface morphology indicative of better compatibility between PI-O and [Bmim]Br. In the film side view, the internal structure of cryofracture films and composites could be observed. The presence of a fibrous surface could be observed with a transversal fiber arrangement in the form of layers, except for pristine PI-O, which shows an irregular globular appearance.

**Figure 6:**
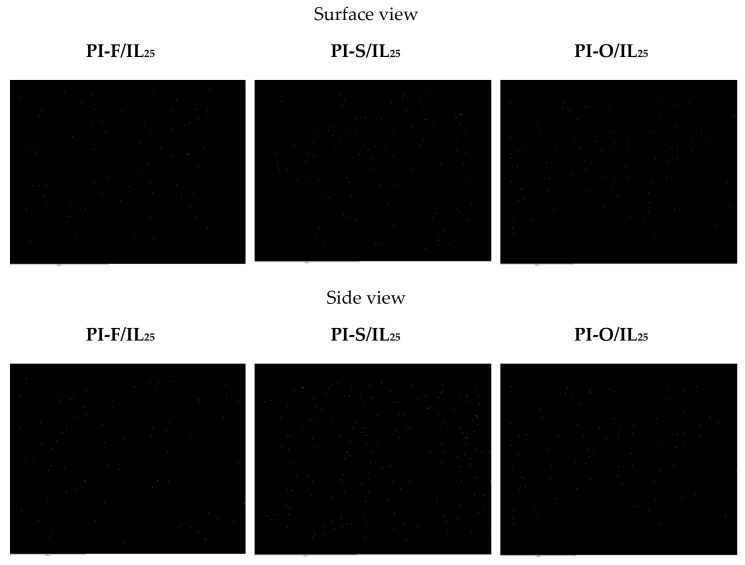
The bright spots in each picture confirm the presence of bromine evenly distributed on the surface and across the film.

The EDX analysis and elemental mappings showed that the distribution of the [Bmim]Br was homogeneous in all films. Figure 6 shows the presence of bromine atoms on the surface and across the films in a random distribution. Therefore, this fact is indicative of good compatibility of the IL within the polymer matrix.

### 3.7. Water Uptake and Contact Angle Measurements

Following the described procedure in the Material and Methods section, the water uptake values for PIs and PI/IL_25_ were obtained from mass difference and expressed as a percentage in Table 5.

The PI films containing oxygen in their central portion (PI-O and PI-S) are able to absorb two or three-fold water amount than poly(imide) containing –C(CF_3_)_2_– groups (PI-F). This result reveals that the number of oxygen atoms on the repeat unit seemed to play an important role in water absorption. This fact suggests that water uptake mechanism could be associated with hydrogen bond formation between those atoms and the hydrogens of the water molecules. Additionally, the presence of ionic liquid in the polymeric matrix increases the ability to absorb water in all samples. In particular, the poly(imide) containing –SO_2_-groups showed the highest water absorption (12 wt%) when the ionic liquid was present. Similar behavior was observed when the experiments were conducted at 80 °C. Under this experimental condition and for all samples, the uptake water was greater than at 30 °C. Despite this increase, the amount of water absorbed for the films were low, which could be attributed to the absence of pores both on the surface and inside them, which is supported by SEM images (Figure 5). It is interesting to note that films having poor water uptake are desirable to reduce dimensional changes under drastic oxidative conditions in the proton exchange membranes [50].

**Figure 7:**
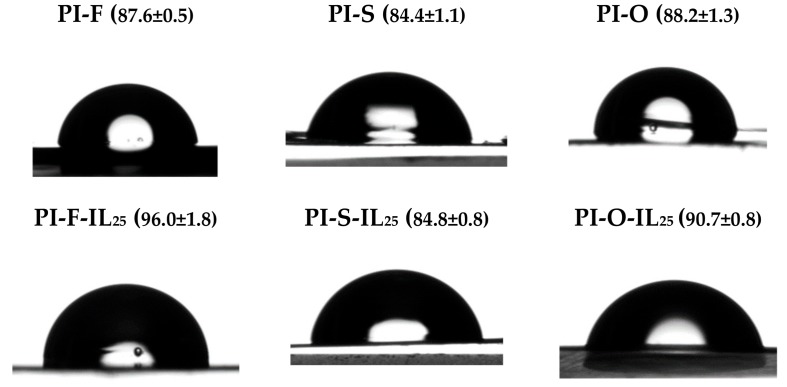
The profiles of a water droplet on the tested films.

The films hydrophobicity was evaluated by measuring the water contact angle (WCA) using the sessile drop method. In Table 5 are summarized the WCA values for PIs and PI/IL_25_ and the respective drop images are shown in Figure 7.

Depending on the contact angle value, a surface could be classified as hydrophilic or hydrophobic. A surface is considered hydrophilic when the contact angle value is lower than 90° and hydrophobic for higher values [51]. The results showed that the films without ionic liquid present a slight hydrophilic character being PI-S the most hydrophilic surface. When [Bmim]Br is loaded into the films, an increase in hydrophobic character was observed in all samples; this is in accordance with the water uptake capacity obtained for each film and the interaction of the ionic liquid with the polar groups inside the polymeric matrix films, leaving polymer hydrophobic zones exposed to the surface. For that reason, the globular agglomerates observed in SEM images could correspond to polymer chains on the surface of the films. This is an important result, because the ionic liquid is responsible for eventual proton conductivity and it must be inside the membrane.

### 3.8. Oxidative Stability

**Figure 8:**
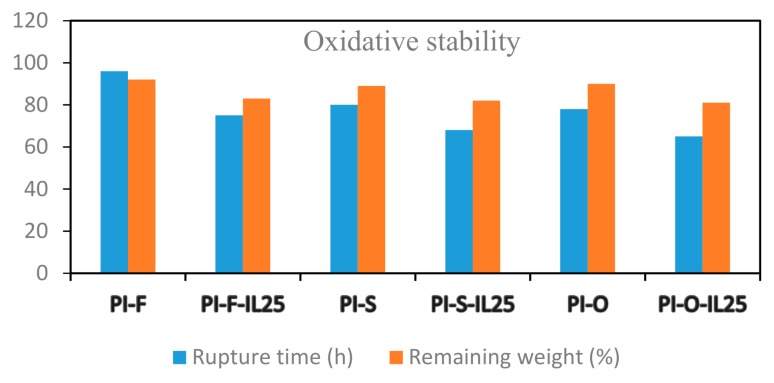
Oxidative stability of the pristine PIs and composed films.

The oxidative stability of the pristine poly(imide) films and those composed with IL was established in accordance with the procedure described in the Material and Methods section. Figure 8 depicts the remaining weights and rupture times of the prepared films.

Films were ruptured between 65–96 h and the weight loss ranged between 8–19%. In general, the polymer oxidative stability was high. In previous research, it was found that introducing heteroatoms groups, like sulfone or fluorine-containing polymers as well as cross-linked polymers, improved the oxidative stability [12,14,52,53,54,55,56,57]. In our case, PI-F displayed a significantly resistant to oxidation and showed lower weight loss than other pristine PI films. In other studies, it has been seen that high radical resistivity of C–F bonds is responsible for polymer stability [58]. Additionally, in all cases, the composed films that contain ionic liquid were less resistant respect to pristine PIs, probably due to degradation of [Bmim]Br by the Fenton’s reagent action [31,59,60]. Due that the degradation was evidenced after two days of analysis; it is possible to affirm again that most of the ionic liquid was incorporated inside the membrane.

## 4. Conclusions

Three new PIs were successfully synthesized via condensation reaction and adequately characterized by spectroscopic techniques. Despite the medium-to-high molecular weight that was suggested by the viscosity measurements, all PIs were soluble in common organic solvent and PI-F was soluble even in THF, a low boiling point solvent. Thermal decomposition temperatures were higher than 512 °C indicating that all PIs were thermally stable, while PI/IL films showed lower T_d5%_ value than PI films due to the presence of IL. From solutions of these polymers and of their respective composite with different content of [Bmim]Br, dense and flexible films were prepared by the casting method. Using PI-F and in accordance with the resistant to handling a maximum content of ionic liquid of 25 wt.% was established. Morphological analysis revealed that inside the films, it is possible to see a similar morphology in all cases showing a fibrous appearance. The water absorption analysis of all films developed at two temperatures was low even in those that contain ionic liquid. The results showed that the presence of ionic liquid in the films increased the hydrophobicity of the surface, proposing specific interactions between the PI and IL. Finally, the presence of the flexible linker in the polymeric backbones showed an excellent film resistance towards radical oxidation during the Fenton test. With these preliminary results, we will continue to study in the future the proton conductivity of the films for their potential application as solid proton exchange devices.

## Figures and Tables

**Table 1:** Reaction conditions for the synthesis of PI-F using LiCl and TMSCl.

Attempt ^a^	TMSCl (mmol)	LiCl (mmol)	DMAc (mL)	η_inh_ (dL/g) ^b^
**1**	-	2	4	0.32
**2**	-	2	8	0.35
**3**	2	-	4	0.43
**4**	2	-	8	0.50
**5**	-	-	8	0.72

^a^ 2 mmol of each monomer was used. ^b^ Inherent viscosity (0.5 g/dL in NMP at 30 °C).

**Table 2:** Solubility and inherent viscosities of poly(imides).

Poly(imide)	η_inh_ (dL/g)^a^	Tested Solvent/Solubility						
		DMSO	DMF	DMAc	NMP	*m*-Cresol	CHCl_3_	THF
**PI-F**	0.72	(+)	(+)	(+)	(+)	(+)*	(−)	(+)
**PI-O**	0.68	(+)	(+)	(+)	(+)	(+)*	(−)	(−)
**PI-S**	0.73	(+)	(+)	(+)	(+)	(+)*	(−)	(−)

^a^ Inherent viscosity (c = 0.5 g/dL in NMP at 30 °C). Solubility: (+) Soluble at room temperature; (+) * Soluble under heating (solvent boiling temperature); (−) Insoluble even after heating.

**Table 3:** [Bmim]Br proportion in PI-F/IL composite films and their mechanical characteristic.

Sample Label	[Bmim]Br (w/w,%)*	Film Quality
**PI-F**	0	Good, resistant to handling
**PI-O**	0	Good, resistant to handling
**PI-S**	0	Good, resistant to handling
**PI-F/IL_10_**	10	Good, resistant to handling
**PI-F/IL_15_**	15	Good, resistant to handling
PI-F/IL_20_	20	Good, resistant to handling
PI-F/IL_25_	25	Good, resistant to handling
PI-F/IL_30_	30	Brittle when the film twists
PI-F/IL_35_	35	Brittle when the film was removed
PI-F/IL_40_	40	Brittle on the Teflon plate

* 500 mg of polymer in each attempt.

**Table 4:** Thermal properties of PIs and PI/IL films a under nitrogen atmosphere.

Sample	*T*_d5%_ (°C) ^a^	*T*_g_ (°C) ^b^	*R*c (%)^c^
PI-F	549	260	58
PI-O	545	225	57
PI-S	512	230	62
PI-F/IL_25_	298	n.d	59
PI-O/IL_25_	281	n.d	60
PI-S/IL_25_	298	n.d	47

^a^ Temperature at 5% weight loss (heating rate of 10 °C/min). ^b^ Glass transition temperature from the second heating run (heating rate 10 °C/min). ^c^ Residual weight after heated at 900 °C. n.d: not determined.

**Table 5:** Water uptake and water contact angle values of the films.

Film *	Water Uptake at 30 °C (wt.%)	Water Uptake at 80 °C (wt.%)	Water Contact Angle (°)
**PI-F**	2.4	3.2	87.6 ± 0.5
**PI-F-IL_25_**	4.4	5.3	96.0 ± 1.8
**PI-S**	8.0	9.1	84.4 ± 1.1
**PI-S-IL_25_**	12.0	13.2	84.8 ± 0.8
**PI-O**	5.3	6.4	88.2 ± 1.3
**PI-O-IL_25_**	9.0	10.1	90.7 ± 0.8

* The film’s thickness was around 64-68 μm.
